# MiR‐145‐5p Attenuates Doxorubicin‐Induced Heart Injury Through Targeting Cardiomyocyte Pyroptosis

**DOI:** 10.1002/kjm2.70126

**Published:** 2025-11-12

**Authors:** Xing‐Tao Chen, Yong‐Hong Yu, Yan‐Hua Du, Lin Xu, Xiang‐Ping Meng

**Affiliations:** ^1^ Department of General Practice Wuhan Fourth Hospital Wuhan China; ^2^ Department of Geriatrics Renmin Hospital of Wuhan University Wuhan China

**Keywords:** cardiotoxicity, doxorubicin, miR‐145‐5p, pyroptosis, SOX9

## Abstract

Doxorubicin (DOX), a potent anthracycline chemotherapeutic, exhibits dose‐dependent cardiotoxicity that limits its clinical utility. Although miR‐145‐5p demonstrates cardioprotective properties in cardiovascular diseases, its role in DOX‐induced cardiomyopathy remains undefined. This study investigated the therapeutic potential of miR‐145‐5p against DOX‐induced cardiotoxicity and its underlying mechanism. Wistar rats received cumulative DOX dosing (15 mg/kg total) to establish cardiotoxicity, with miR‐145‐5p overexpression achieved via adeno‐associated virus serotype 9 (AAV9) delivery. Cardiac function was assessed by echocardiography and serum biomarkers, including creatine kinase‐MB isoenzyme (CK‐MB), cardiac troponin T (c‐TnT), C‐reactive protein (CRP), and N‐terminal pro‐B‐type natriuretic peptide (NT‐proBNP). Histopathology (Hematoxylin & Eosin/Masson's Trichrome staining), apoptosis (TUNEL), oxidative stress (dihydroethidium staining/malondialdehyde/glutathione), and NLRP3 inflammasome activation (ELISA/Western blot/immunohistochemistry) were evaluated. Clinical relevance was determined by quantifying serum miR‐145‐5p and SOX9 mRNA in healthy controls and breast cancer patients before and after DOX treatment. DOX significantly downregulated miR‐145‐5p and upregulated SOX9 in rat myocardium and H9C2 cells. DOX‐treated patients displayed reduced serum miR‐145‐5p and increased SOX9 mRNA compared with pre‐chemotherapy baselines. AAV9‐miR‐145‐5p attenuated DOX‐induced systolic dysfunction, reduced serum biomarkers, ameliorated histopathological injury and fibrosis, suppressed apoptosis and oxidative stress, and inhibited NLRP3 inflammasome activation (decreased NLRP3, ASC, caspase‐1, IL‐1β, and IL‐18). miR‐145‐5p directly targeted the SOX9 3′UTR, and SOX9 overexpression reversed miR‐145‐5p–mediated reductions in CK release, ROS production, apoptosis, and NLRP3 expression in H9C2 cells. These findings demonstrate that miR‐145‐5p protects against DOX cardiotoxicity by targeting SOX9 to inhibit NLRP3 inflammasome–mediated pyroptosis, offering a potential therapeutic strategy.

## 1 Introduction

The anthracycline doxorubicin (DOX) has been widely used to treat various human cancers [1]. However, its clinical utility is constrained by dose‐dependent cardiotoxicity, which may progress to heart failure in long‐term cancer survivors [2]. Dexrazoxane remains the only US Food and Drug Administration (FDA)‐approved cardioprotectant for anthracycline chemotherapy, yet it carries risks of bone marrow suppression, hepatotoxicity, and secondary malignancies [3]. Therefore, developing safe adjuvant therapies for DOX‐induced cardiotoxicity is clinically imperative.

The mechanisms underlying DOX cardiotoxicity involve mitochondrial dysfunction, oxidative stress, ferroptosis, dysregulated autophagy, inflammation, and apoptosis [4], with reactive oxygen species (ROS) overproduction as a central event [5]. In cardiomyocytes, the quinone structure of DOX undergoes reduction to semiquinone radicals, increasing oxygen consumption and generating excessive ROS [6]. This oxidative cascade damages lipids, proteins, and DNA, compromising membrane integrity and triggering mitochondrial collapse that culminates in apoptosis [2]. Additionally, caspase‐dependent pyroptosis contributes significantly via activation of nucleotide‐binding oligomerization domain (NOD)‐like receptors, particularly the NOD‐like receptor family pyrin domain containing 3 (NLRP3) inflammasome. This complex proteolytically activates interleukin (IL)‐1β and IL‐18, amplifying cardiac inflammation [7]. DOX‐induced cardiotoxicity is mechanistically linked to NLRP3‐mediated pyroptosis, with ROS serving as a key trigger for inflammasome assembly in cardiomyocytes [8]. Thus, targeting ROS–NLRP3 signaling represents a promising therapeutic strategy.

MicroRNAs (miRNAs) regulate gene expression through mRNA degradation or translational repression, modulating ~30% of human genes [9]. They critically influence physiological and pathological processes [10], including cardiovascular disease. MicroRNA‐145‐5p (miR‐145‐5p) exhibits cardioprotective properties: its downregulation correlates with chronic heart failure severity [11], its overexpression attenuates angiotensin II‐induced hypertrophy [12], and it mitigates ischemic injury by suppressing apoptosis [13]. However, its role in DOX‐induced cardiotoxicity remains unclear.

SRY‐box transcription factor 9 (SOX9), a transcription factor involved in sex determination and chondrogenesis [14], also influences cardiac pathophysiology. Cardiac‐specific SOX9 deletion inhibits ischemic hypertrophy [15], while its silencing protects against hypoxic apoptosis by suppressing caspase‐3/9 activation [16]. Critically, SOX9 knockdown reduces NLRP3‐driven pyroptosis in septic cardiomyopathy [17], but its involvement in DOX‐induced cardiotoxicity has not been defined.

This study investigates the therapeutic potential of miR‐145‐5p against DOX‐induced cardiotoxicity and examines whether its cardioprotective effects occur via regulation of SOX9/NLRP3 inflammasome signaling, providing mechanistic insights for the development of novel treatment strategies.

## 2 Materials and Methods

### 2.1 Clinical Sample Collection

Serum samples were prospectively collected from two cohorts at Wuhan Fourth Hospital between January 2023 and December 2023 under Institutional Review Board approval: (1) healthy control cohort: 10 age‐ and gender‐matched volunteers (58.3 ± 6.2 years, 5 male/5 female) with normal cardiac function (left ventricular ejection fraction [LVEF] > 55%, N‐terminal pro‐B‐type natriuretic peptide [NT‐proBNP] < 125 pg/mL), and no history of malignancy, cardiovascular disease, or renal/hepatic dysfunction; (2) DOX‐exposed cohort: 10 diagnosed stage II–III breast cancer patients (60.1 ± 5.8 years, 4 male/6 female) scheduled to receive ≥ 3 cycles of DOX‐based chemotherapy (cumulative dose ≥ 150 mg/m^2^). Exclusion criteria included prior anthracycline exposure, concurrent radiotherapy, autoimmune disorders, or elevated baseline troponin T (> 14 ng/L). For patients, venous blood (5 mL) was obtained at two time points: (i) baseline (24 h before the first chemotherapy cycle) and (ii) 48 h after the final DOX infusion. Samples were processed within 30 min by centrifugation at 3000 × *g* for 15 min at 4°C, aliquoted into RNase/DNase‐free cryovials, and stored at −80°C until miRNA/mRNA analysis. Written informed consent was obtained from all participants.

### 2.2 Animals

Specific pathogen‐free male Wistar rats (180–210 g, 8 weeks old) were obtained from Beijing Beiyou Biotechnology Co. Ltd. (Beijing, China). Animals were housed individually in ventilated polycarbonate cages under controlled environmental conditions (temperature 22°C ± 1°C, humidity 50% ± 10%, 12‐h light/dark cycle) with *ad libitum* access to food and water and acclimatized for 7 days before experimentation. All procedures complied with the eighth edition of the Guide for the Care and Use of Laboratory Animals (National Institutes of Health, 2011) and were approved by the Animal Ethics Committee of the Wuhan Fourth Hospital.

### 2.3 DOX‐Induced Cardiotoxicity Model

Rats were randomly assigned to four groups (*n* = 10 per group): (1) NS + AAV9‐cTNT‐Scramble (NS + miR‐NC), saline‐treated rats receiving a tail vein injection of adeno‐associated virus serotype 9 (AAV9) encoding a scrambled miRNA under the cardiac troponin T (cTNT) promoter (Hanheng Biotechnology, Shanghai, China); (2) NS + AAV9‐cTNT‐miR‐145‐5p (NS + miR‐145‐5p), saline‐treated rats receiving AAV9 expressing miR‐145‐5p under the cTNT promoter; (3) DOX + AAV9‐cTNT‐Scramble (DOX + miR‐NC), rats injected with AAV9‐cTNT‐scramble 14 days before the first DOX dose; and (4) DOX + AAV9‐cTNT‐miR‐145‐5p (DOX + miR‐145‐5p), rats injected with AAV9‐cTNT‐miR‐145‐5p 14 days before the first DOX dose. AAV9‐mediated cardiac miRNA delivery is a well‐established preclinical strategy, with proven efficacy and cardiac tropism demonstrated in prior studies of heart failure [18], ischemic injury [19], and hypertrophy [20]. DOX (Product No. ajci18230, Amgicam, Wuhan, China) was administered via intraperitoneal injection at 2.5 mg/kg three times a week for 2 weeks (cumulative dose: 15 mg/kg). This cumulative dosing regimen is a well‐established model for DOX‐induced chronic cardiotoxicity, effectively mimicking clinical cardiotoxicity and reliably inducing significant cardiac injury/dysfunction, as supported by references [21, 22, 23]. AAV9‐Scramble or AAV9‐miR‐145‐5p was delivered via a single tail vein injection (200 μL volume) at 5 × 10^11^ vg/rat [20] 14 days before initial DOX exposure. All injections used sterile 0.9% saline (NS) as vehicle.

### 2.4 Cell Culture and Transfection

H9C2 rat cardiomyoblast cells (CM‐0089, Procell, Wuhan) were maintained in Dulbecco's Modified Eagle's Medium (DMEM; Gibco C11995500BT) supplemented with 10% fetal bovine serum (Gibco 10270‐106) and antibiotics (100 U/mL penicillin and 100 μg/mL streptomycin; Gibco 15140‐122) at 37°C in a humidified incubator with 5% CO_2_ and 95% air. For transfection experiments, cells were seeded in 6‐well plates (5 × 10^5^ cells/well) and grown to 70%–80% confluence prior to transfection. The pcDNA3.1 empty vector (GenePharma, Shanghai, China), pcDNA3.1‐SOX9 overexpression plasmid (GenePharma), miR‐145‐5p mimics, miR‐145‐5p inhibitor, negative control (NC) mimics, and NC inhibitor were transfected at 80 nM final concentration using Lipofectamine 2000 transfection reagent (11668030, Invitrogen) following manufacturer protocols. Complexes were incubated with cells for 6 h in serum‐free DMEM, after which the medium was replaced with complete growth medium. All subsequent experiments were conducted 48 h post‐transfection to ensure adequate gene expression modulation.

### 2.5 Cell Viability Measurement

H9C2 cells were seeded in 96‐well plates at 5 × 10^3^ cells/well and incubated overnight. Cells were then exposed to DOX at concentrations of 1, 3, 5, 7, and 10 μM for 24 h. After treatment, 10 μL 3‐(4,5‐dimethylthiazol‐2‐yl)‐2,5‐diphenyltetrazolium bromide (MTT; 5 mg/mL; SJ1074, Carnoss, Wuhan) was added per well and incubated for 4 h at 37°C. The supernatant was carefully aspirated, and 150 μL DMSO (D2650, Sigma‐Aldrich) was added to dissolve formazan crystals. Absorbance was measured at 490 nm using a FLUOstar Omega multimode microplate reader (BMG LABTECH, Ortenberg, Germany).

### 2.6 Flow Cytometry Analysis

H9C2 cells were seeded in 6‐well plates at 1 × 10^6^ cells/well and incubated overnight. After treatment, cells were harvested using 0.25% trypsin‐Ethylenediaminetetraacetic Acid (EDTA) without phenol red (Gibco 25200072), washed twice with cold phosphate‐buffered saline (PBS), and resuspended in 1× Annexin V binding buffer. Apoptosis was evaluated using an Annexin V‐FITC/PI Apoptosis Detection Kit (640914, BioLegend, Beijing) following the manufacturer's protocol: 5 μL fluorescein isothiocyanate (FITC)‐conjugated Annexin V and 5 μL propidium iodide (PI) working solution were added to 195 μL cell suspension, followed by a 15‐min incubation at room temperature in the dark. Samples were analyzed immediately using a NovoCyte 3000 flow cytometer (Agilent Technologies), with 10,000 events acquired per sample. Data were analyzed using NovoExpress 1.6.1 software.

### 2.7 Detection of ROS

In vivo ROS levels in rat heart tissue were assessed using dihydroethidium (DHE) staining. Fresh cardiac tissues were embedded in optimal cutting temperature compound, snap‐frozen in liquid nitrogen, and stored at −80°C. Cryosections (10 μm thickness) were fixed in 4% paraformaldehyde (P6148, Sigma–Aldrich) for 15 min, washed with PBS, and incubated with 10 μM DHE (D7008, Sigma–Aldrich) at 37°C for 30 min in the dark. Sections were counterstained with 4′,6‐diamidino‐2‐phenylindole (DAPI; C0065, Solarbio, Beijing) and mounted. Fluorescence images were captured using a Zeiss Axio Imager fluorescence microscope. Quantification was performed by analyzing ≥ 5 random fields per section (≥ 3 sections per animal) using ImageJ software. DHE fluorescence intensity (red) was normalized to the DAPI‐stained area (blue) with background subtraction and expressed as fold change relative to the control group (set as 1.0). In vitro ROS levels in H9C2 cells were measured using 2′,7′‐Dichlorodihydrofluorescein diacetate (DCFH‐DA; CA1410, Solarbio). After incubation with 10 μM DCFH‐DA (37°C, 25 min), fluorescence intensity was visualized under a Zeiss 800 confocal microscope and quantified by flow cytometry. Results represent the mean fluorescence intensity relative to the control group (set as 1.0) per 10,000 cells.

### 2.8 Echocardiography

Following anesthesia induction with 5% isoflurane/95% O_2_ and maintenance at 1.5% isoflurane, rats were positioned in the left lateral decubitus position on a heated platform (37°C). Cardiac function was assessed using a Vevo 3100 high‐resolution ultrasound system (FUJIFILM VisualSonics) with an MX550D transducer (40 MHz center frequency; 40 μm axial resolution). Parasternal long‐axis M‐mode tracings at the papillary muscle level (depth: 25–30 mm; frame rate: 200–300 fps) recorded three consecutive cardiac cycles. Left ventricular internal dimensions at end‐diastole (LVIDd) and end‐systole (LVIDs) were measured across five cardiac cycles by two blinded investigators. Left ventricular fractional shortening (LVFS) was calculated as [(LVIDd−LVIDs)/LVIDd] × 100%, and LVEF was derived via the Teichholz method using VevoLAB v5.6.1 software.

### 2.9 Enzyme‐Linked Immunosorbent Assay (ELISA)

LV blood samples (1 mL) collected using EDTA‐coated tubes were centrifuged at 3000 × *g* for 15 min at 4°C to separate serum, which was aliquoted and stored at −80°C. Serum levels of creatine kinase‐MB isoenzyme (CK‐MB), c‐TnT, C‐reactive protein (CRP), and NT‐proBNP were quantified using commercial ELISA kits: CK‐MB ELISA Kit (CSB‐E14403r, CUSABIO, Wuhan), c‐TnT ELISA Kit (CSB‐E16443r, CUSABIO), CRP ELISA Kit (CSB‐E07922r, CUSABIO), and NT‐proBNP ELISA Kit (BES2802K, BIOSEN, Shanghai). All assays followed manufacturers' protocols, and measurement was performed using a FLUOstar Omega multimode microplate reader at 450 nm. For myocardial tissue cytokines, 50 mg LV tissue was homogenized in 500 μL RIPA lysis buffer (P0013B, Beyotime, Shanghai), centrifuged at 12,000 × *g* (4°C, 20 min), and supernatants were assayed undiluted for IL‐1β using ELISA Kit (ml037361, mlbio, Shanghai) and IL‐18 using ELISA Kit (PI555, Beyotime), with optical density normalized to total protein quantified by bicinchoninic acid (BCA) assay (23227, Thermo Scientific).

### 2.10 Detection of Oxidative Stress Markers

Malondialdehyde (MDA) content and reduced glutathione (GSH) levels in LV tissue were quantified using commercial kits: MDA detection was performed with the Lipid Peroxidation MDA Assay Kit (S0131S, Beyotime) based on thiobarbituric acid reactive substances (TBARS) methodology where homogenates (10% w/v in PBS) reacted with 0.67% thiobarbituric acid at 95°C for 90 min before absorbance measurement at 532 nm; GSH was measured via the Glutathione Colorimetric Detection Kit (K261, Biovision) utilizing Ellman's reagent (5,5′‐dithiobis‐2‐nitrobenzoic acid) which forms yellow 5‐thio‐2‐nitrobenzoic acid upon reaction with thiol groups with absorbance read at 412 nm, both assays using supernatants from heart tissue homogenates centrifuged at 12,000 × *g* (4°C, 20 min) and normalized to protein concentration determined by BCA assay with results expressed as nmol MDA/mg protein and μmol GSH/g tissue, respectively.

### 2.11 Histopathological Analysis

Following euthanasia, rat hearts were immediately harvested and fixed in 10% neutral buffered formalin (G2161, Solarbio) for 48 h, processed through graded ethanol dehydration, xylene clearing, and paraffin embedding. Sections (5 μm thickness) were stained with Hematoxylin & Eosin (H&E; G1120, Solarbio) for histopathological assessment. Adjacent sections underwent Masson's Trichrome staining (G1340, Solarbio) with standardized protocol: Weigert's iron hematoxylin (10 min), Biebrich scarlet‐acid fuchsin (5 min), and aniline blue (5 min). Histological evaluation was performed by two blinded cardiovascular pathologists who analyzed ≥ 8 non‐overlapping fields per section (≥ 3 sections/animal) using a Nikon Eclipse Ti2 microscope. Collagen volume fraction was quantified in ImageJ 1.53k by thresholding aniline blue‐positive areas relative to total tissue area across all fields, with data expressed as fold‐change relative to controls (set as 1.0).

### 2.12 Immunohistochemistry

Paraffin‐embedded cardiac sections (5 μm) underwent deparaffinization in xylene (10 min × 3), rehydration through graded ethanol (100% → 70%), and epitope retrieval via microwave heating in 0.01 M citrate buffer (pH 6.0, 95°C, 20 min). Endogenous peroxidase activity was quenched with 0.5% hydrogen peroxide for 30 min, followed by blocking with 3% bovine serum albumin (A8020, Solarbio) for 1 h at room temperature. Sections were incubated overnight at 4°C with primary rabbit polyclonal anti‐NLRP3 antibody (DF15549, Affinity; 1:50). After washing, sections were incubated with HRP‐conjugated goat anti‐rabbit secondary antibody (A0208, Beyotime; 1:200) for 1 h, then visualized with 3,3′‐diaminobenzidine (DA1010, Solarbio) chromogen substrate (5‐min reaction). All sections were counterstained with hematoxylin (1 min), dehydrated, and mounted for brightfield microscopy (Nikon Eclipse Ti2). For quantification, ≥ 8 non‐overlapping fields per section were captured. NLRP3‐positive area (brown staining) was measured via ImageJ 1.53k color thresholding and normalized to total tissue area. Data were expressed as fold‐change relative to the control group (set as 1.0).

### 2.13 Terminal‐Deoxynucleoitidyl Transferase‐Mediated Nick End Labeling (TUNEL) Staining

Cardiac tissue was washed in ice‐cold PBS, fixed in 4% paraformaldehyde for 24 h at 4°C, dehydrated through graded ethanol (70% → 100%), cleared in xylene, and paraffin embedded. Five‐micrometer sections underwent deparaffinization/rehydration before permeabilization with 0.1% Triton X‐100 (T8200, Solarbio) in PBS for 15 min. TUNEL staining was performed using the TUNEL Apoptosis Detection Kit (MK1026, BOSTER Biotech, Wuhan) following the manufacturer's protocol. Nuclei were counterstained with DAPI for 10 min. Sections were mounted in antifade medium (P0126, Beyotime) and imaged with confocal microscopy (Zeiss 800). TUNEL^+^ nuclei were quantified as a percentage of total DAPI^+^ nuclei in ≥ 10 random fields per heart using ZEN 3.1 software. Flow cytometry analysis was conducted on collagenase‐dissociated cardiomyocytes stained identically, quantifying TUNEL^+^/DAPI^+^ populations in the NovoCyte 3000 flow cytometer.

### 2.14 Quantitative Real‐Time Polymerase Chain Reaction (RT‐qPCR)

Total RNA was extracted from rat LV tissue, H9C2 cells, and 200 μL human serum samples using TRIzol LS reagent (10296028, Invitrogen) for serum and TRIzol (15596026, Invitrogen) for tissue/cells, with serum samples spiked with 5 fmol synthetic cel‐miR‐39 (219610, Qiagen) prior to extraction for normalization. cDNA synthesis used 1 μg total RNA (or 10 ng serum RNA) with the RevertAid First Strand cDNA Synthesis Kit (K1621, Thermo Scientific) for mRNAs (65°C/5 min → 42°C/60 min) and the miRNA First Strand Synthesis Kit (CW2141, CWBio, Beijing) for miRNAs (37°C/60 min) per manufacturer protocols. RT‐qPCR was performed on a QuantStudio 6 Flex System (Applied Biosystems) using PowerUp SYBR Green Master Mix (A25742, Applied Biosystems) in triplicate 20‐μL reactions (95°C/10 min; 40 cycles: 95°C/15 s → 60°C/60 s) with melting curve analysis (65°C–95°C). The primers used are as follows: miR‐145‐5p (F: 5′‐GTCCAGTTTTCCCAGGAATCCCT‐3′, R: 5′‐GCTGTCAACATACGCTACGTAACG‐3′), SOX9 (F: 5′‐AAAGGAAGGAAGGGAAGAAAG‐3′, R: 5′‐AATATGGCATCT TTCGATTTCTG‐3′), U6 snRNA (F: 5′‐CTCGCTTCGGCAGCACA‐3′, R: 5′‐AACGCTTCACGAATTTGCGT‐3′), GAPDH (F: 5′‐GCAAGTTCAACGGCACAG‐3′, R: 5′‐GCCAGTAGACTCCACGACAT‐3′). Serum miRNA data were normalized to cel‐miR‐39 spike‐in, while tissue/cell data to U6 (miRNAs) or GAPDH (mRNAs), all analyzed by the 2^−ΔΔCt^ method.

### 2.15 Western Blot

Protein extracts from LV tissue and H9C2 cells were prepared in RIPA lysis buffer with homogenization/centrifugation (12,000 × *g*, 20 min, 4°C) and quantified using the BCA assay. Equal protein aliquots (30 μg) underwent separation on 10% sodium dodecyl sulfate‐polyacrylamide gel electrophoresis, followed by electrophoretic transfer to polyvinylidene fluoride membranes (IPVH00010, Millipore). Membranes were blocked with 5% non‐fat milk (P0216, Beyotime) for 2 h at room temperature before overnight incubation at 4°C with primary antibodies: SOX9 (AF6330, Affinity; 1:500), NLRP3 (DF7438, Affinity; 1:500), apoptosis‐associated speck‐like protein containing CARD (ASC) (DF6304, 1:1000), caspase‐1 (AF5418, Affinity; 1:500), IL‐1β (AF5103, Affinity; 1:1000), IL‐18 (DF6252, Affinity; 1:500), and GAPDH (AF7021, Affinity; 1:3000). After washing, membranes were incubated with HRP‐conjugated goat anti‐rabbit IgG (S0006, Affinity; 1:500) for 1 h at room temperature, developed using Ultra‐sensitive enhanced chemiluminescence reagent (DY30208, DEEYEE, Shanghai), and visualized on a ChemiDoc XRS+ imaging system (Bio‐Rad). Band intensity was quantified by densitometry using ImageLab 6.1 software (Bio‐Rad), normalized to GAPDH.

### 2.16 Luciferase Reporter Assay

The putative miR‐145‐5p binding site within the 3′UTR of rat SOX9 was cloned into the pmirGLO Dual‐Luciferase Vector (E1330, Promega). The wild‐type sequence was mutated to generate mutant SOX9 (SOX9‐Mut) constructs using the Q5 Site‐Directed Mutagenesis Kit (E0554S, New England BioLabs). H9C2 cells seeded in 24‐well plates (1.5 × 10^4^ cells/well) were co‐transfected at 70% confluency using Lipofectamine 2000 with 10 ng pmirGLO‐SOX9‐Wt or pmirGLO‐SOX9‐Mut reporter plasmid +20 nM miR‐145‐5p mimics or NC mimics. Luciferase activity was quantified 48 h post‐transfection using the Dual‐Luciferase Reporter Assay System (E1910, Promega).

### 2.17 Safety and Tissue Distribution Assessment of AAV9‐miR‐145‐5p

Male Wistar rats (8 weeks old) were divided into two groups: Normal control (*n* = 8) receiving tail vein injection of 200 μL saline, and AAV9‐miR‐145‐5p (*n* = 8) receiving AAV9 vector (5 × 10^11^ vg/rat) expressing miR‐145‐5p under the cTNT promoter via tail vein. Fourteen days post‐injection, serum was collected for biochemical analysis of alanine aminotransferase (ALT), aspartate aminotransferase (AST), alkaline phosphatase (ALP), blood urea nitrogen (BUN), creatinine (Cr), and lactate dehydrogenase (LDH) using automated clinical chemistry analyzers, while heart, liver, kidney, lung, and brain tissues were harvested with subsamples either snap‐frozen for TRIzol‐based RNA extraction followed by RT‐qPCR quantification of miR‐145‐5p (normalized to U6 snRNA) or fixed in 10% neutral buffered formalin for paraffin embedding, sectioning at 5 μm thickness, and standard H&E staining to assess histopathological integrity across all sampled organs.

### 2.18 Statistical Analysis

All quantitative data were analyzed using GraphPad Prism 9.0.0. Continuous variables are presented as mean ± standard deviation with individual data points shown in scatter plots. Pearson correlation analysis was performed to assess linear relationships. Between‐group comparisons employed: Unpaired two‐tailed Student's *t*‐test for two independent groups; One‐way analysis of variance with Bonferroni correction for multigroup comparisons. Statistical significance was defined as *p* < 0.05. Sample sizes (*n*) represent biological replicates detailed in figure captions.

## 3 Results

### 3.1 MiR‐145‐5p is Downregulated and SOX9 is Upregulated in DOX‐Induced Cardiotoxicity

**FIGURE 1:**
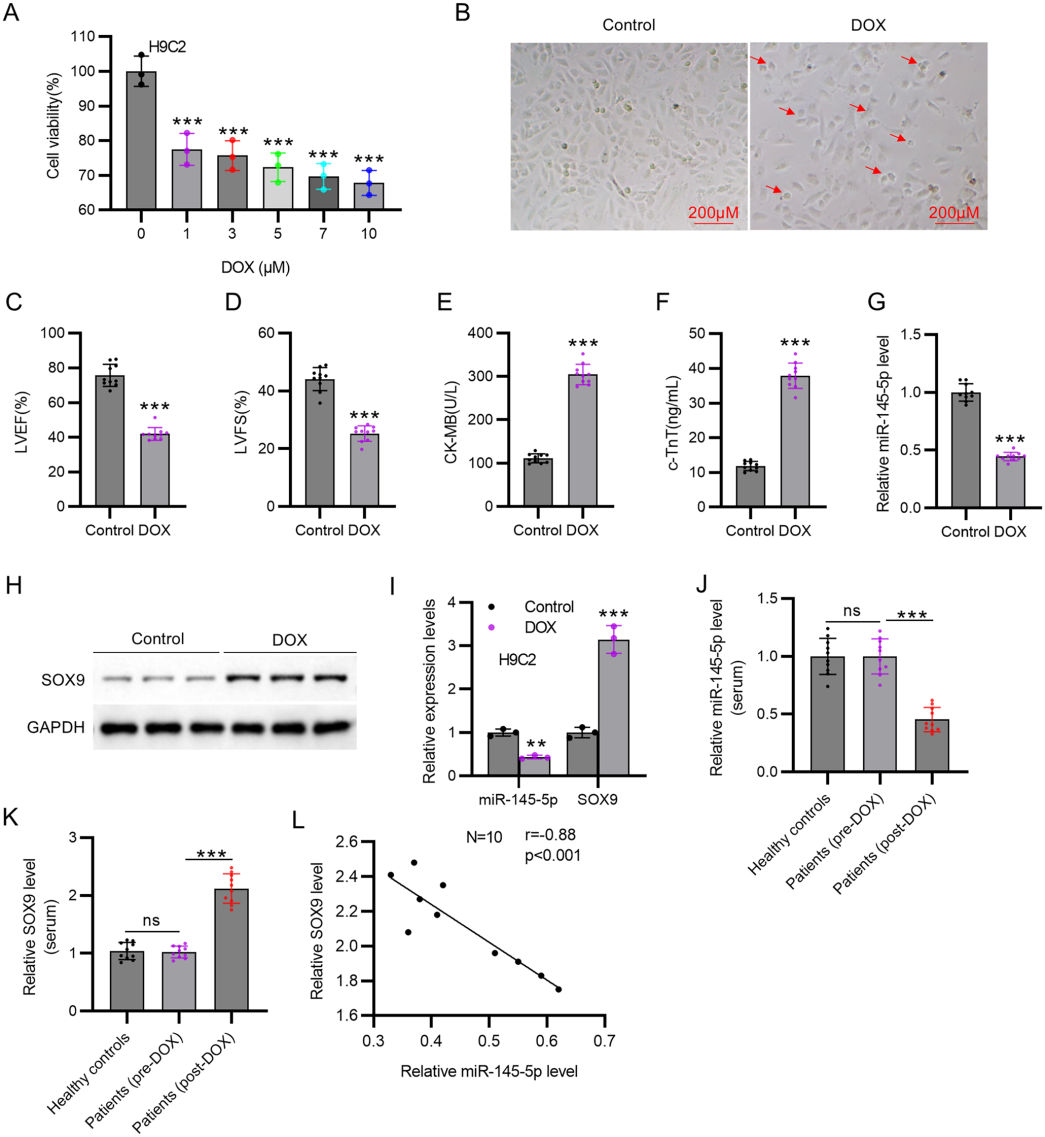
MiR‐145‐5p is downregulated and SOX9 is upregulated in DOX‐induced cardiotoxicity. (A) Viability of H9C2 cells treated with DOX (1, 3, 5, 7, and 10 μM) for 24 h detected by MTT. *N* = 3 individual experiments. (B) The morphology of H9C2 cells treated with 5 μM DOX for 24 h observed by light microscopy (scale bar = 200 μm). Arrows indicate cell shrinkage. *N* = 3 individual experiments. (C) LVEF and (D) LVFS in Control group and DOX group. *N* = 10 animals per group. (E) Serum CK‐MB and (F) c‐TnT levels in Control group and DOX group. *N* = 10 animals per group. (G) MiR‐145‐5p expression in rat hearts of Control group and DOX group detected by RT‐qPCR. *N* = 10 animals per group. (H) SOX9 protein expression in control group and DOX group measured by Western blot. *N* = 10 animals per group. (I) MiR‐145‐5p (mRNA) and SOX9 (mRNA) expression levels in DOX‐treated (5 μM, 24 h) H9C2 cells detected by RT‐qPCR. *N* = 3 individual experiments. (J) Serum miR‐145‐5p levels and (K) SOX9 mRNA levels measured by RT‐qPCR in healthy controls, cancer patients before DOX chemotherapy (pre‐DOX), and cancer patients 48 h after final DOX infusion (post‐DOX). *N* = 10 per group. (L) Correlation analysis of miR‐145‐5p and SOX9 levels in 10 DOX‐treated patients. *N* = 10. Data are presented as the mean ± standard deviation. ***p < 0.001.

MTT assay revealed that different concentrations of DOX (1, 3, 5, 7, and 10 μM) significantly reduced the viability of H9C2 cells (*p* < 0.001, Figure 1A). As shown in Figure 1B, 5 μM DOX significantly reduced the number of H9C2 cells and changed cell morphology. A rat model of DOX‐induced cardiotoxicity was established. LVEF and LVFS were significantly reduced in DOX‐treated rats compared to saline‐treated rats (*p* < 0.001, Figure 1C,D). The serum levels of CK‐MB and c‐TnT (myocardial injury markers) were also increased in the DOX group compared to the control group (*p* < 0.001, Figure 1E,F). DOX treatment markedly reduced miR‐145‐5p mRNA expression and elevated SOX9 protein expression in rat hearts (*p* < 0.001, Figure 1G,H). Additionally, miR‐145‐5p mRNA expression was downregulated and SOX9 mRNA expression was upregulated in DOX‐treated H9C2 cells (*p* < 0.001, Figure 1I). Analysis of serum samples revealed that DOX chemotherapy significantly altered circulating biomarker profiles. Compared to healthy controls, miR‐145‐5p levels were unchanged in pre‐chemotherapy cancer patients but sharply decreased by 53.9% in the same cohort 48 h post‐final DOX infusion (*p* < 0.001, Figure 1J). Conversely, SOX9 mRNA levels exhibited a reciprocal 112% increase post‐treatment while remaining unaltered at baseline (Figure 1K). This inverse correlation (Figure 1L) suggests miR‐145‐5p may regulate SOX9 in human DOX cardiotoxicity.

### 3.2 MiR‐145‐5p Alleviates DOX‐Induced Cardiac Injury

**FIGURE 2:**
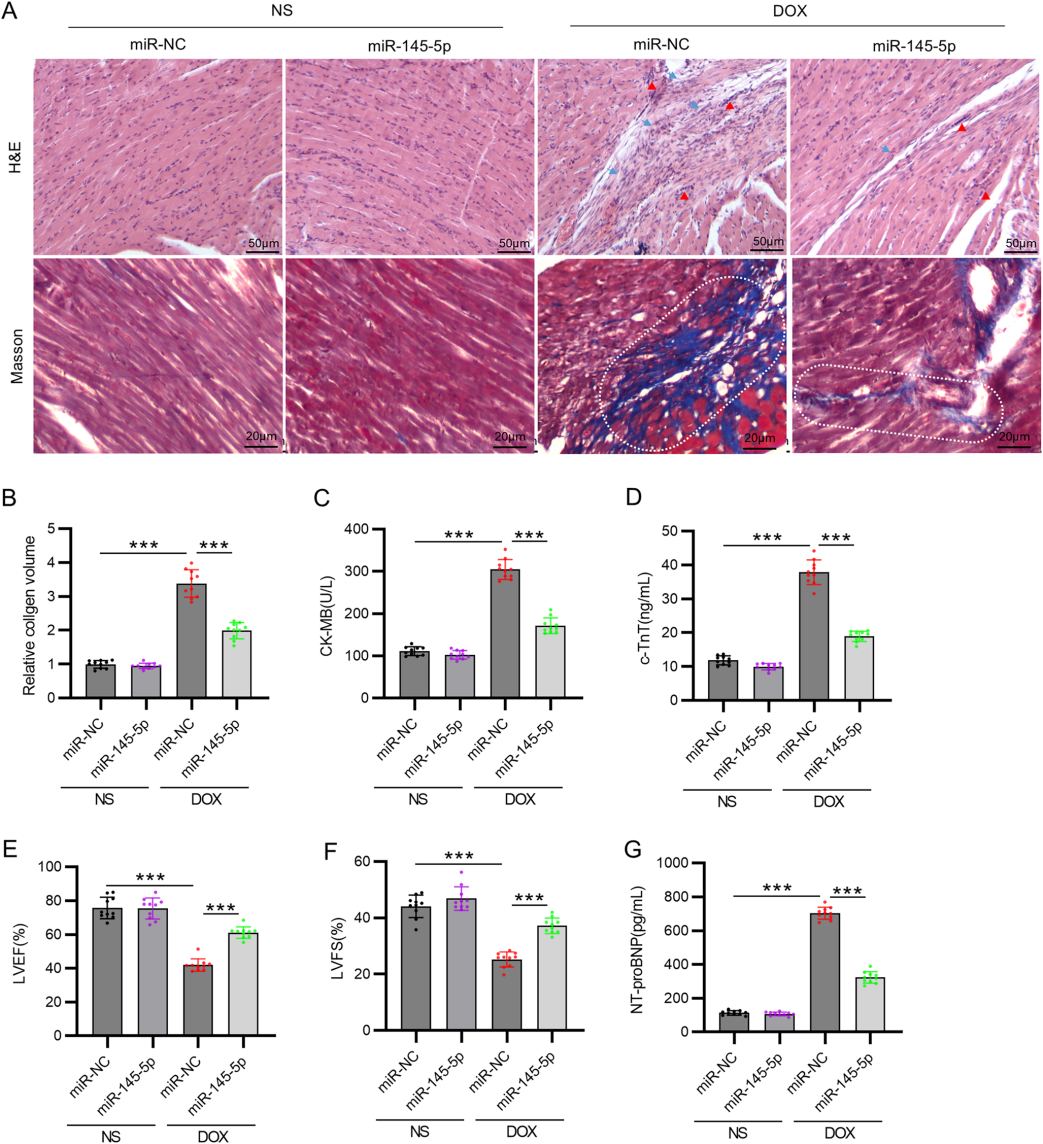
MiR‐145‐5p overexpression alleviates DOX‐induced cardiac injury. (A) A representative cardiac tissue sections stained with H&E (scale bar = 50 μm) and Masson's Trichrome (scale bar = 20 μm). Histopathological annotations: (arrow) cardiomyocyte degeneration; (triangle) inflammatory cell infiltration; (rectangle) fibrotic collagen deposition (blue in Masson). (B) Quantification of collagen volume. (C) Serum CK‐MB and (D) c‐TnT levels. (E) LVEF and (F) LVFS. (G) Quantification of serum NT‐proBNP levels. *N* = 10 animals per group. Data are presented as the mean ± standard deviation. ***p < 0.001.

H&E and Masson staining showed that, in the absence of DOX, the hearts in the AAV9‐miR‐145 group had no significant change compared to the miR‐NC group. However, under the stimulation of DOX, the hearts exhibited obvious intermuscular edema, cardiomyocyte degeneration, inflammatory cell infiltration, and fibrosis. All these pathological changes were mitigated by the injection of AAV9‐miR‐145‐5p (*p* < 0.001, Figure 2A,B). In comparison to the control group, there was an increase in CK‐MB and c‐TnT levels in the DOX group (*p* < 0.001), which were restored after overexpression of miR‐145‐5p (*p* < 0.001, Figure 2C,D). Furthermore, the injection of AAV9‐miR‐145‐5p significantly improved LVEF and LVFS in DOX‐treated rats (*p* < 0.001, Figure 2E,F). Consistent with impaired systolic function, serum NT‐proBNP, a gold‐standard biomarker for heart failure, was markedly elevated in DOX rats versus controls (*p* < 0.001). miR‐145‐5p overexpression attenuated this increase by 53.4% (*p* < 0.001, Figure 2G), confirming functional improvement at the molecular level.

### 3.3 MiR‐145‐5p Inhibits DOX‐Induced Cardiomyocyte Apoptosis and Oxidative Stress

**FIGURE 3:**
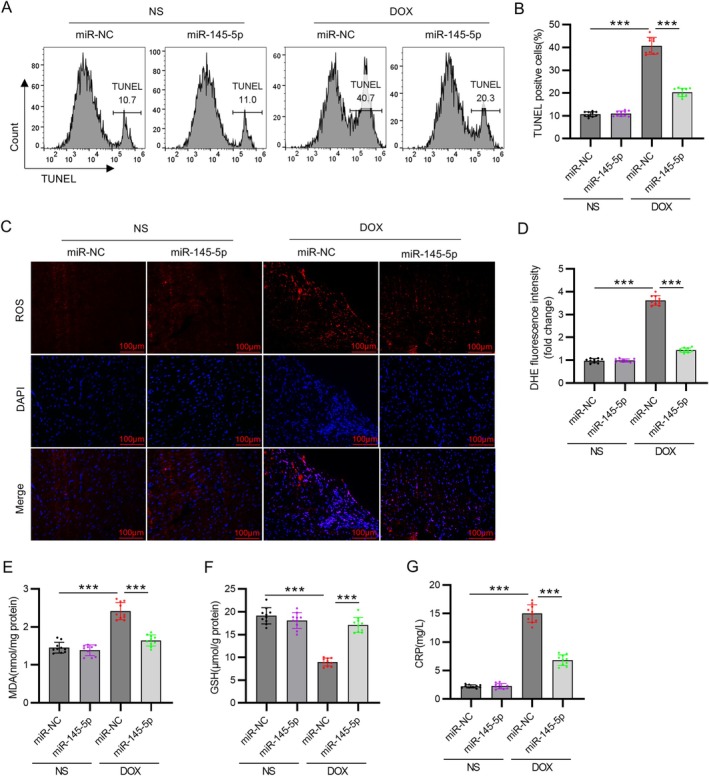
MiR‐145‐5p overexpression inhibits DOX‐induced cardiomyocyte apoptosis and oxidative stress. (A) Flow cytometry analysis of TUNEL staining. (B) Quantification of TUNEL positive cells. (C) Representative images of cardiac tissue sections stained with DHE (scale bar = 100 μm). (D) Quantification of ROS production. (E) MDA and (F) GSH levels in rat hearts. (G) Quantification of serum CRP levels. *N* = 10 animals per group. Data are presented as the mean ± standard deviation. ***p < 0.001.

TUNEL staining indicated that the DOX group presented an increased number of apoptotic cells compared to normal controls. However, the injection of AAV9‐miR‐145‐5p markedly decreased cardiomyocyte apoptosis induced by DOX (*p* < 0.001, Figure 3A,B). The DHE staining results revealed that DOX administration increased ROS levels, which were reversed by overexpression of miR‐145‐5p (*p* < 0.001, Figure 3C,D). In the DOX group, MDA levels were increased and GSH levels were decreased compared to the control group (*p* < 0.001). Overexpression of miR‐145‐5p significantly restored the levels of MDA and GSH in DOX‐treated rats (*p* < 0.001, Figure 3E,F). Systemic inflammation was quantified via serum CRP, which showed a significant increase in DOX rats versus controls (*p* < 0.001). This elevation was reduced by 53% with miR‐145‐5p intervention (*p* < 0.001, Figure 3G), mechanistically linking oxidative stress to inflammatory response.

### 3.4 MiR‐145‐5p Restrains DOX‐Induced Activation of SOX9‐NLRP3 Inflammasome

**FIGURE 4:**
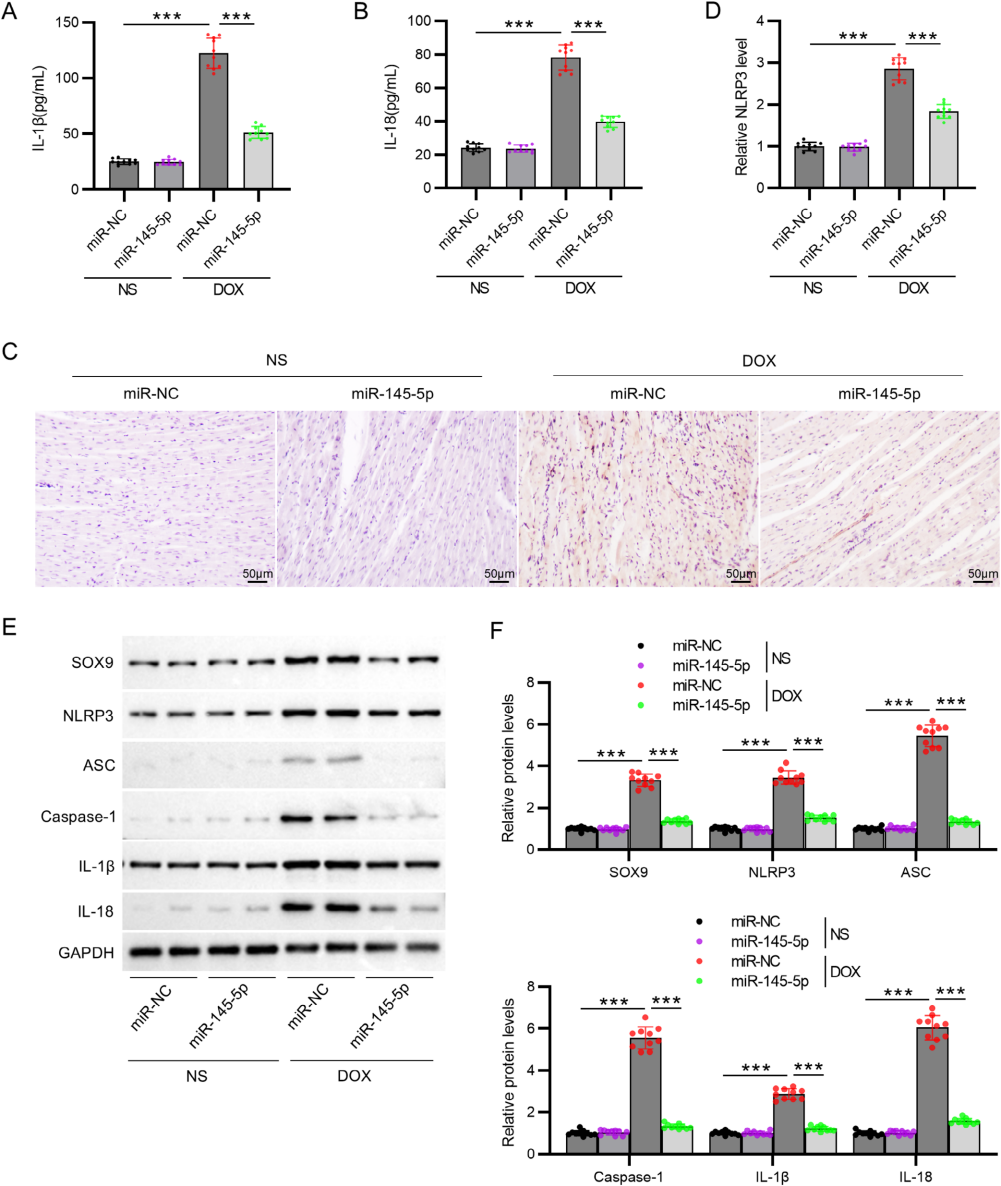
MiR‐145‐5p inhibits DOX‐induced activation of SOX9‐NLRP3 inflammasome. (A) IL‐1β and (B) IL‐18 levels in rat hearts. (C, D) NLRP3 protein localization in cardiac tissue sections by immunohistochemistry (scale bar = 50 μm). (E) Representative immunoblots of SOX9 and NLRP3 inflammasome components in rat hearts. (F) Densitometric quantification of protein expression. *N* = 10 animals per group. Data are presented as the mean ± standard deviation. ***p < 0.001.

To explore the miR‐145‐5p's effect on cardiomyocyte pyroptosis, we measured the levels of pyroptosis‐related proteins in rat models. The IL‐1β and IL‐18 levels in rat hearts were markedly increased in the DOX group compared to the control group (*p* < 0.001), which were restored by the injection of AAV9‐miR‐145‐5p (*p* < 0.001, Figure 4A,B). Immunohistochemistry showed that the injection of AAV9‐miR‐145‐5p reversed the increase in NLRP3 expression in DOX‐treated rats (*p* < 0.001, Figure 4C,D). Moreover, Western blot showed that miR‐145‐5p overexpression reduced the protein levels of SOX9, NLRP3, ASC, caspase‐1, IL‐1β, and IL‐18 in DOX‐treated rats (*p* < 0.001, Figure 4E,F).

### 3.5 Safety Profile and Tissue‐Specific Expression of AAV9‐miR‐145‐5p

Blood biochemistry analysis demonstrated comparable levels of liver function markers (ALT, AST, ALP), kidney function markers (BUN, Cr), and general tissue damage marker (LDH) between Normal and AAV9‐miR‐145‐5p groups (all *p* > 0.05, Figure S1A–F), confirming no hepatorenal toxicity. Tissue‐specific expression analysis revealed exclusive cardiac enrichment of miR‐145‐5p in AAV9‐treated rats (12.6‐fold vs. normal heart, *p* < 0.001), with minimal changes in non‐target organs (liver: 1.1‐fold, kidney: 1.0‐fold, lung: 1.2‐fold, brain: 1.0‐fold) (Figure S1G). Representative H&E staining showed preserved cytoarchitecture in heart, liver, kidney, and lung tissues of both groups, with no evidence of necrosis, inflammatory infiltration, or structural abnormalities (Figure S1H), further validating the biosafety of AAV9‐mediated cardiac delivery.

### 3.6 SOX9 is Targeted by miR‐145‐5p

**FIGURE 5:**
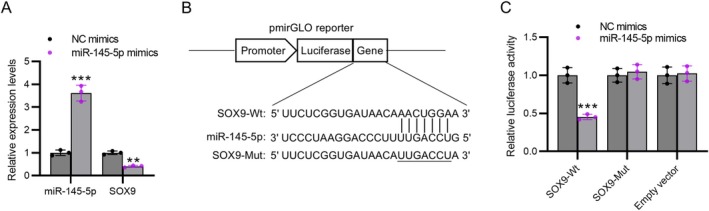
SOX9 is targeted by miR‐145‐5p. (A) MiR‐145‐5p (mRNA) and SOX9 (mRNA) expression in H9C2 cells transfected with miR‐145‐5p mimics or NC mimics detected by RT‐qPCR. (B) Predicted miR‐145‐5p binding site (wild‐type) and engineered mutant site in SOX9 3′UTR. (C) Luciferase reporter activity in H9C2 cells co‐transfected with wild‐type SOX9 3′UTR plasmid (SOX9‐Wt), mutant SOX9 3′UTR plasmid (SOX9‐Mut), empty vector, and miR‐145‐5p mimics or NC mimics. *N* = 3 individual experiments. Data are presented as the mean ± standard deviation. **p < 0.01, ***p < 0.001.

The RT‐qPCR data indicated that transfection with miR‐145‐5p mimics increased miR‐145‐5p mRNA expression and reduced SOX9 mRNA expression in H9C2 cells (*p* < 0.01 or *p* < 0.001, Figure 5A), suggesting that miR‐145‐5p negatively regulates SOX9. TargetScan 7.1 predicted that SOX9 has a miR‐145‐5p binding site in its 3′UTR (Figure 5B). Luciferase reporter assay was performed to validate this interaction, showing that miR‐145‐5p overexpression reduced the luciferase activity of SOX9 3′UTR‐Wt plasmids (*p* < 0.001) but did not affect that of SOX9 3′UTR‐Mut plasmids (Figure 5C), demonstrating that miR‐145‐5p targets SOX9.

### 3.7 SOX9 Overexpression Reverses the Effect of miR‐145‐5p on Cardiomyocyte Injury

**FIGURE 6:**
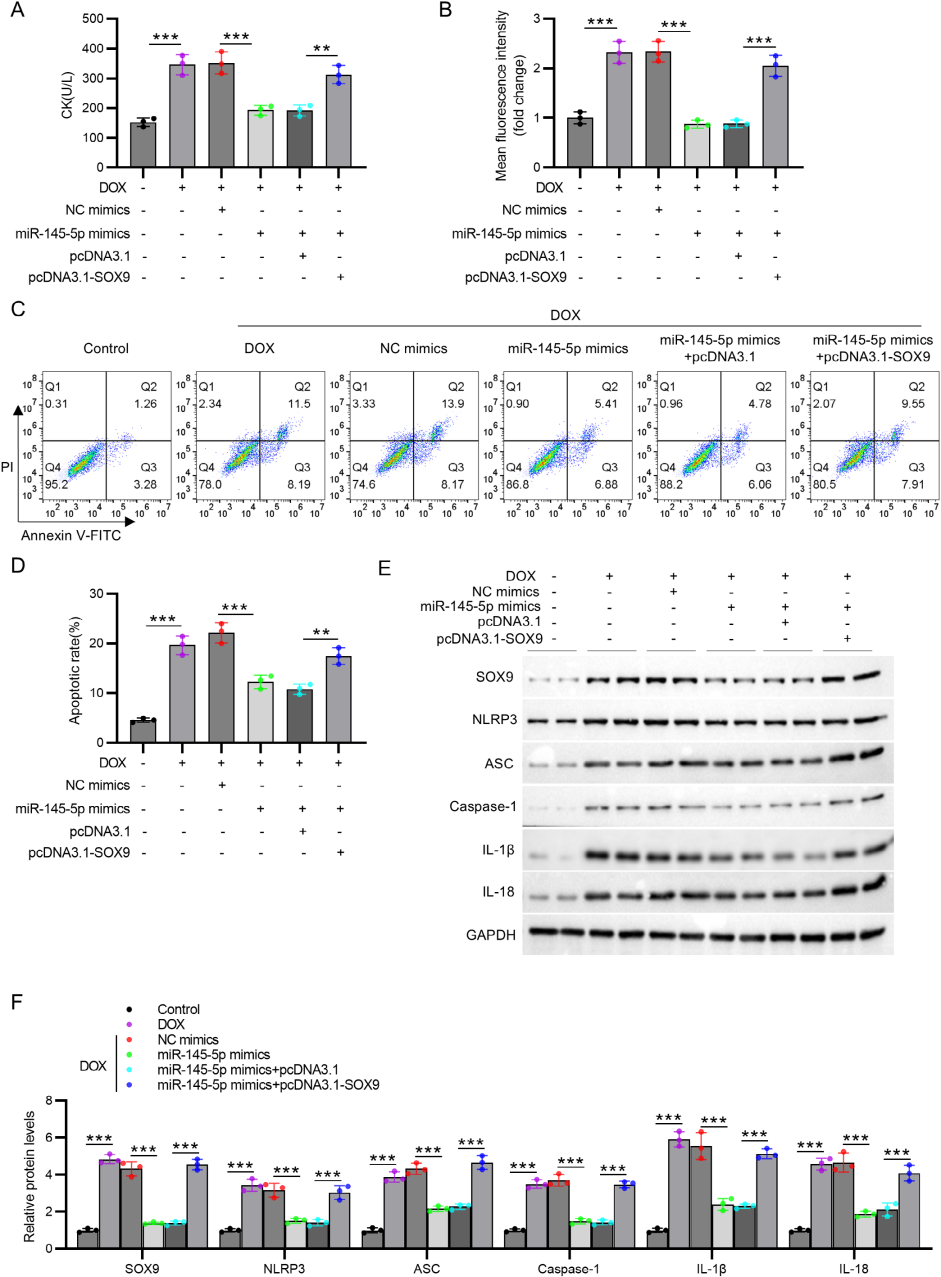
SOX9 overexpression reverses the effect of miR‐145‐5p against cardiomyocyte injury. (A) CK release measured by ELISA. (B) ROS production assessed using DCFH‐DA followed by flow cytometry. (C) Representative flow cytometry plots of Annexin V/PI staining. (D) Quantification of apoptotic cells. (E) Representative immunoblots of SOX9 and NLRP3 inflammasome components. (F) Densitometric quantification of protein expression. *N* = 3 individual experiments. Data are presented as the mean ± standard deviation. **p < 0.01, ***p < 0.001.

**FIGURE 7:**
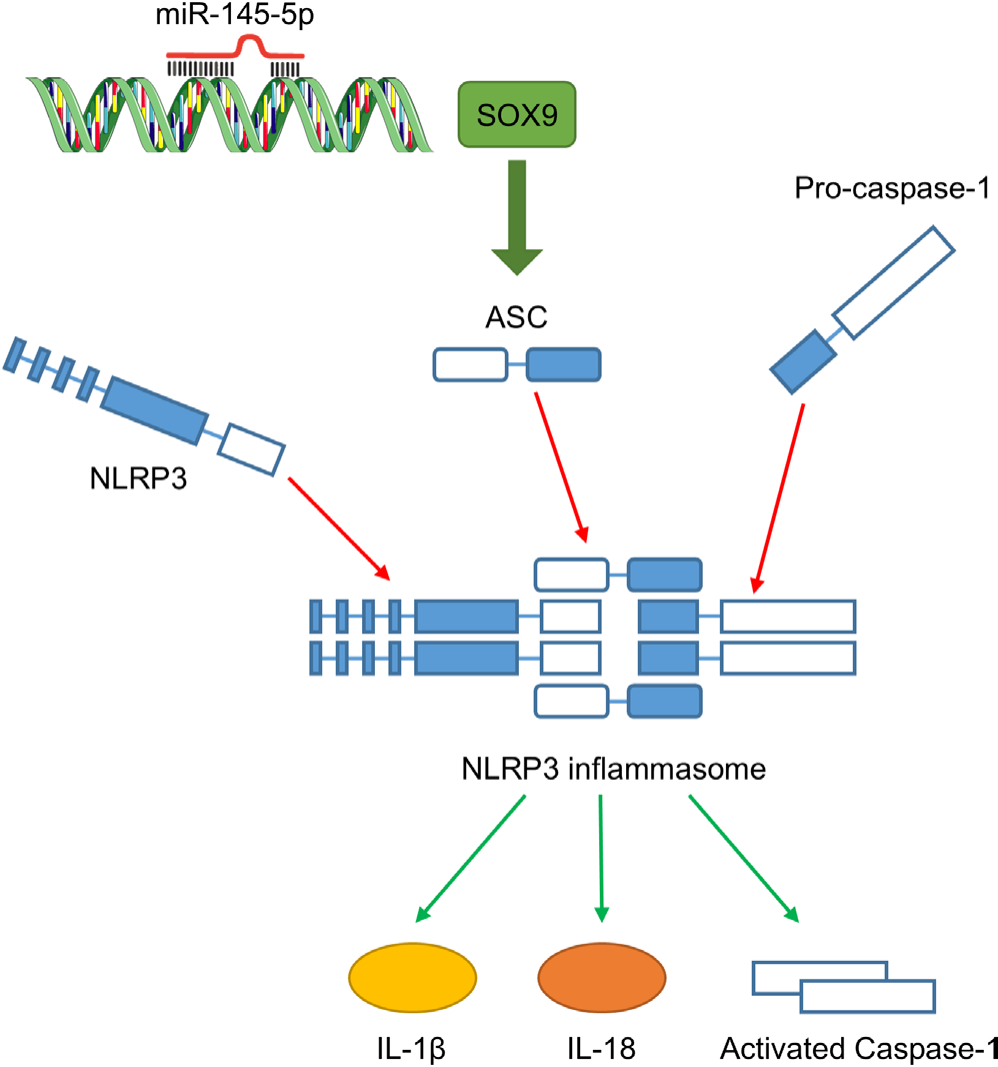
Mechanism underlying the effect of miR‐145‐5p against DOX‐induced cardiotoxicity.

To verify the mechanisms, we performed in vitro rescue experiments. The results showed that miR‐145‐5p overexpression markedly reduced CK levels, ROS production, and apoptosis in DOX‐treated H9C2 cells, but this effect was reversed by SOX9 overexpression (*p* < 0.01 or *p* < 0.001, Figure 6A–D). Moreover, miR‐145‐5p overexpression reversed DOX‐induced increases in SOX9, NLRP3, ASC, caspase‐1, IL‐1β, and IL‐18 levels in H9C2 cells, which were reversed by SOX9 overexpression (*p* < 0.001, Figure 6E,F), suggesting that miR‐145‐5p alleviates DOX‐induced cardiomyocyte injury by inhibiting the SOX9‐mediated NLRP3 inflammasome pathway (Figure 7).

## 4 Discussion

In recent years, miRNAs have been considered as promising biomarkers and therapeutic targets for DOX‐induced cardiotoxicity [24]. Our findings reveal that miR‐145‐5p, which is significantly downregulated in the serum of DOX‐treated breast cancer patients, exerts potent cardioprotection in vivo. Specifically, AAV9‐mediated overexpression of miR‐145‐5p alleviated DOX‐induced cardiotoxicity in rats by concurrently reducing oxidative stress and suppressing NLRP3 inflammasome‐mediated pyroptosis.

Pyroptosis is a crucial inflammatory caspase‐dependent programmed cell death, which is involved in inflammasome activation and proinflammatory effects [25]. As the widely characterized inflammasome, the NLRP3 inflammasome can recruit adapter protein ASC and pro‐caspase‐1 to promote the activation of caspase‐1. Activated caspase‐1 subsequently shifts pro‐IL‐1β and pro‐IL‐18 to mature IL‐1β and IL‐18, leading to the release of proinflammatory cytoplasmic contents [26]. Studies have shown that persistent activation of the NLRP3 inflammasome leads to adverse consequences, including cardiac dysfunction and remodeling [27]. Zhang et al. reported that MCC950, an NLRP3 inflammasome inhibitor, alleviated DOX‐induced myocardial injury through suppressing NLRP3‐mediated pyroptosis [28]. Inhibition of pyroptosis‐related molecules, such as NLRP3, caspase‐1, and Bnip3, may represent a strategy to limit DOX‐induced cardiotoxicity [29]. In DOX‐induced cardiac injury, DOX could induce pyroptosis to promote myocardial fibrosis and cardiac remodeling, leading to the deterioration of cardiac function [30]. Several studies have demonstrated the inhibitory effect of miR‐145‐5p on NLRP3 inflammasome activation [31, 32]. Consistent with previous studies, we also found that miR‐145‐5p overexpression downregulated the levels of pyroptosis‐related molecules including NLRP3, ASC, caspase‐1, IL‐1β, and IL‐18. Our data support the hypothesis that miR‐145‐5p overexpression inhibits pyroptosis, thereby protecting against DOX cardiotoxicity.

Previous studies have demonstrated the antioxidant effect of miR‐145‐5p in heart disease. Li et al. reported that overexpression of miR‐145‐5p inhibited apoptosis and reduced inflammatory responses and oxidative stress in hypoxia/reoxygenation‐treated cardiac microvascular endothelial cells [33]. Liang et al. reported that miR‐145‐5p played a protective role against myocardial ischemia injury by alleviating oxidative stress [34]. It is well known that overproduction of ROS is a key influencing factor that can induce heart failure, cardiac arrhythmia, and other pathological states [35]. Moreover, DOX‐induced cardiotoxicity is mainly driven by ROS‐mediated oxidative stress. DOX promotes the production of a large amount of ROS by interfering with the mitochondrial electron transport chain and activating NADPH oxidase, leading to DNA damage, protein oxidation, and lipid peroxidation [36]. In addition, ROS activates the NLRP3 inflammasome by oxidizing mitochondrial DNA (mtDNA) or mitochondrial outer membrane lipids and releasing DAMP‐related molecular patterns (DAMPs). ROS‐induced DAMPs bind to NLRP3 to promote the recruitment of ASC, thereby activating caspase‐1 and promoting the release of inflammatory factors [37]. Therefore, the development of therapeutic strategies targeting ROS and the NLRP3 inflammasome (such as ROS scavengers or NLRP3 inhibitors) may provide new avenues for the prevention and treatment of DOX‐induced cardiotoxicity. Our data showed that miR‐145‐5p overexpression significantly reduced DOX‐induced ROS production, inflammation, and cell death, supporting the critical role of ROS and the NLRP3 inflammasome in DOX‐induced cardiotoxicity. Future studies will further explore the target genes and signaling pathways of miR‐145‐5p, especially how it regulates ROS production and NLRP3 inflammasome activation. Critically, in vivo delivery of miR‐145‐5p via AAV9 conferred multi‐dimensional cardiac protection against DOX toxicity, as evidenced by preserved systolic function (LVEF/LVFS), reduced myocardial injury markers (CK‐MB/c‐TnT), attenuated pathological remodeling (fibrosis quantification), suppressed cardiomyocyte death (TUNEL/DHE indices), diminished heart failure biomarker (NT‐proBNP), and interrupted oxidative‐inflammatory cascades (MDA/GSH/CRP). This comprehensive protection mechanistically originates from miR‐145‐5p's dual blockade of the SOX9/NLRP3 signaling axis and ROS amplification network, substantiating its capacity to halt the progression from initial oxidative injury to terminal heart failure.

We identified significantly elevated SOX9 expression in both DOX‐treated rats and H9C2 cells, establishing its dual utility as a mechanistic driver and promising biomarker for DOX cardiotoxicity. Critically, this biomarker potential was reinforced by an increase in serum SOX9 mRNA levels in DOX‐treated breast cancer patients versus pre‐chemotherapy baselines. SOX9 plays a pivotal regulatory role in cardiovascular pathogenesis [38], wherein its downregulation mitigates oxidative stress and inflammation in ischemic injury models [39]. Importantly, SOX9 is essential for NLRP3‐mediated pyroptosis, as demonstrated by its capacity to exacerbate sepsis‐induced cardiac damage through NLRP3 upregulation [17]. Our study mechanistically links SOX9 to DOX cardiotoxicity: SOX9 overexpression abolished miR‐145‐5p‐mediated suppression of NLRP3 inflammasome activation and cardiomyocyte pyroptosis. Furthermore, SOX9 restored DOX‐induced ROS accumulation in miR‐145‐5p‐overexpressing H9C2 cells, likely through transcriptional regulation of ROS‐generating genes (e.g., NOX4 activation via NADPH oxidase). Collectively, these findings position SOX9 as a central regulator of DOX cardiotoxicity by orchestrating ROS‐dependent NLRP3 inflammasome activation.

There are several limitations in this study: (1) The study was based on a specific cumulative DOX dose (15 mg/kg) and a single AAV‐miR‐145‐5p titer, which limits insight into potential dose‐dependent effects and the overall therapeutic range. Evaluating dose–response relationships is an important goal for future translational studies; (2) While our clinical validation confirmed dysregulation of the miR‐145‐5p/SOX9 axis in DOX‐treated patients, the study was limited to serum biomarkers at a single post‐treatment timepoint. Future longitudinal studies with multiple sampling timepoints and direct cardiac tissue analysis are warranted to define dynamic changes during cardiotoxicity progression; (3) Although acute biosafety of AAV9 delivery was established through biochemistry and histopathology, comprehensive metabolic impacts (e.g., cytochrome P450 interactions) and whole‐body pharmacokinetics (vector persistence/clearance) require future characterization; (4) The biodistribution analysis focused on miRNA expression without assessing vector genome kinetics, necessitating chronic model studies with quantitative autoradiography.

## 5 Conclusion

In conclusion, this study reveals that miR‐145‐5p confers cardioprotection against DOX‐induced cardiotoxicity by targeting SOX9 to inhibit NLRP3 inflammasome‐mediated pyroptosis, thereby rescuing impaired cardiac contractility, attenuating pathological remodeling, and ultimately mitigating heart failure progression. These findings establish miR‐145‐5p as both a therapeutic target and a potential biomarker, with its ability to preserve systolic function and suppress key drivers of cardiac decompensation offering a novel molecular strategy for preventing DOX‐induced functional decline in clinical oncology.

## Ethics Statement

The animal experiments were performed following the Guide for the Care and Use of Laboratory Animals, and the experimental protocol was approved by the Animal Ethics Committee of Wuhan Fourth Hospital.

## Conflicts of Interest

The authors declare no conflicts of interest.

## Supporting information


**Figure S1:** Safety profile and tissue‐specific expression of AAV9‐miR‐145‐5p. (A–F) Serum levels of hepatic enzymes (ALT, AST, ALP), renal function markers (BUN, Cr), and lactate dehydrogenase (LDH) in Normal control rats and AAV9‐miR‐145‐5p‐treated rats. (G) RT‐qPCR quantification of miR‐145‐5p expression in heart, liver, kidney, lung, and brain tissues from both groups. (H) Representative H&E‐stained sections of heart, liver, kidney, and lung tissues from Normal and AAV9‐miR‐145‐5p‐treated rats. *N* = 8 animals per group. Data are presented as the mean ± standard deviation. ***p < 0.001.

## Data Availability

The data that support the findings of this study are available from the corresponding author upon reasonable request.
