# Supplementary figures and images for: MiR‐145‐5p Attenuates Doxorubicin‐Induced Heart Injury Through Targeting Cardiomyocyte Pyroptosis
Source: Kaohsiung J Med Sci. 2025 Nov 12;42(5):e70126. doi: 10.1002/kjm2.70126 (PMC13182607; doi:10.1002/kjm2.70126)

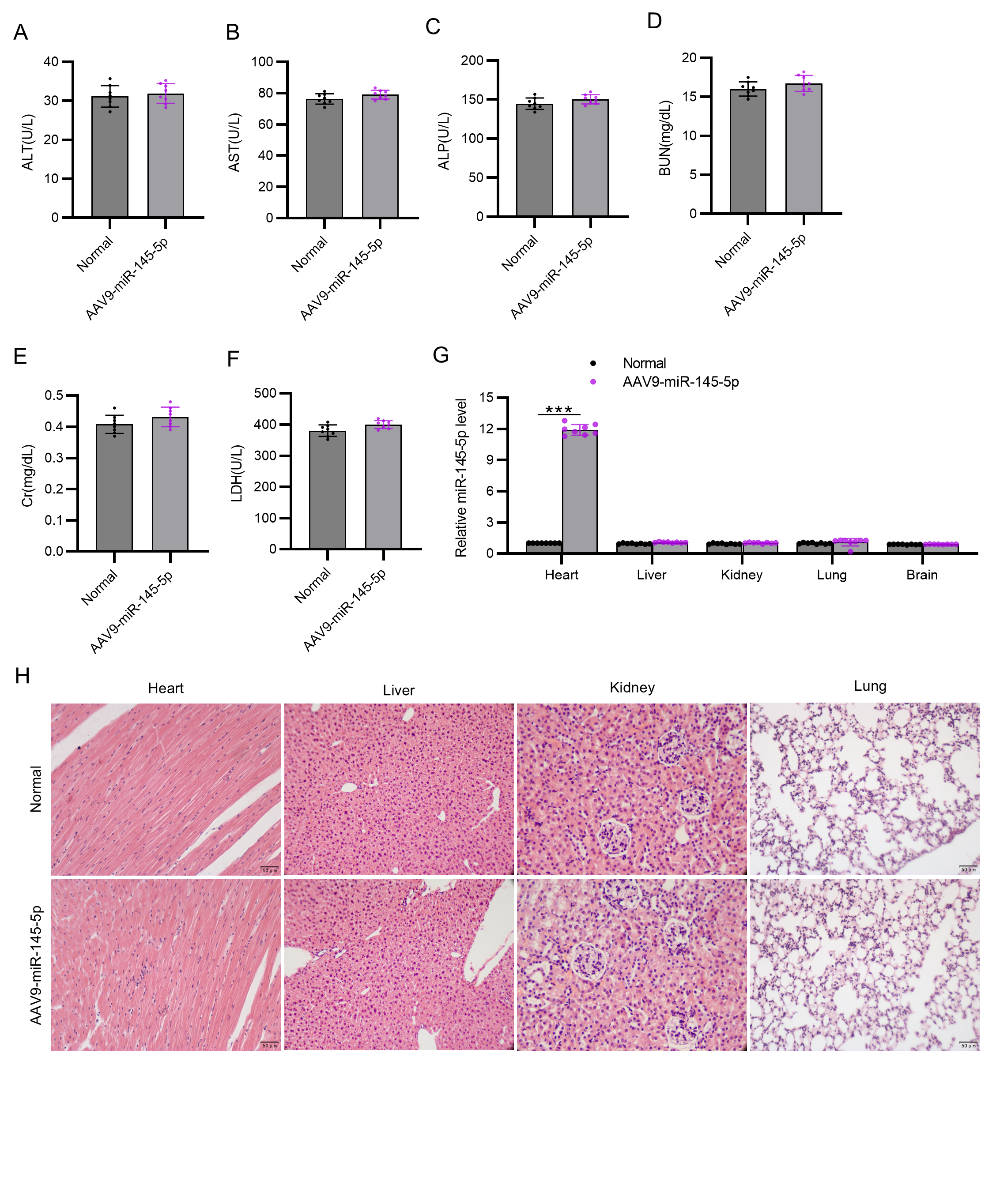

Supplement: Supplementary file 1 — Figure S1: Safety profile and tissue‐specific expression of AAV9‐miR‐145‐5p. (A–F) Serum levels of hepatic enzymes (ALT, AST, ALP), renal function markers (BUN, Cr), and lactate dehydrogenase (LDH) in Normal control rats and AAV9‐miR‐145‐5p‐treated rats. (G) RT‐qPCR quantification of miR‐145‐5p expression in heart, liver, kidney, lung, and brain tissues from both groups. (H) Representative H&E‐stained sections of heart, liver, kidney, and lung tissues from Normal and AAV9‐miR‐145‐5p‐treated rats. N = 8 animals per group. Data are presented as the mean ± standard deviation. ***p < 0.001. [file KJM2-42-e70126-s001.tif]
